# Profiling renal sodium transporters in mice with nephron Ift88 disruption: Association with sex, cysts, and blood pressure

**DOI:** 10.14814/phy2.15206

**Published:** 2022-03-11

**Authors:** Chunyan Hu, Jayalakshmi Lakshmipathi, Deborah Stuart, Donald E. Kohan

**Affiliations:** ^1^ Division of Nephrology University of Utah Health Salt Lake City Utah USA

**Keywords:** Ift88, Na^+^ transport, nephron, sex

## Abstract

Loss of nephron primary cilia due to disruption of the *Ift88* gene results in sex‐ and age‐specific phenotypes involving renal cystogenesis, blood pressure (BP) and urinary Na^+^ excretion. Previous studies demonstrated that male mice undergoing induction of nephron‐specific *Ift88* gene disruption at 2 months of age developed reduced BP and increased salt‐induced natriuresis when pre‐cystic (2 months post‐induction) and became hypertensive associated with frankly cystic kidneys by 9 months post‐induction; in contrast, female Ift88 KO mice manifested no unique phenotype 2 months post‐induction and had mildly reduced BP 9 months post‐induction. The current study utilized these Ift88 KO mice to investigate associated changes in renal Na^+^ transporter and channel protein expression. At 2 months post‐induction, pre‐cystic male Ift88 KO mice had reduced high salt diet associated total NKCC2 levels while female mice had no alterations in Na^+^ transporters or channels. At 9 months post‐induction, cystic male Ift88 KO mice had increased total and phosphorylated NHE3 levels together with reduced NKCC2, phosphorylated and/or total NCC, and ENaC‐α expression on normal and high salt diets. In contrast, female Ift88 KO mice at 9 months post‐induction had no changes in Na^+^ transporters or channels beyond an increase in phosphorylated‐NCC during high salt intake. Thus, reduced BP in pre‐cystic, and elevated BP in renal cystic, male Ift88 KO mice are associated with unique sex‐dependent changes in nephron Na^+^ transporter/channel expression.

## Graphical abstract

We found that NKCC2 is downregulated in male mice lacking nephron cilia; this is associated with relatively low blood pressure as previously reported. In addition, we found that NHE3 is unregulated in male mice lacking cilia who have developed polycystic kidneys; this is associated with hypertension as previously reported.

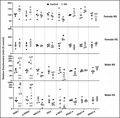

## 1 INTRODUCTION

Defects in primary cilia structure and/or function can cause polycystic kidney disease (PKD) in mice. Animals with inactivating mutations of intraflagellar transport protein 88 (Ift88), a critical ciliary component, have been widely studied (Hovater et al., 2008; Liu et al., 2005; Siroky et al., 2006; Yoder, 2007). *Ift88* gene disruption during embryogenesis or in the first few weeks postnatal causes rapidly progressive and severe PKD, while *Ift88* gene knockout (KO) after 1–2 months of age causes delayed PKD onset by several months (Davenport et al., 2007; Lehman et al., 2008). We previously induced nephron specific *Ift88* gene KO (Ift88 KO) in mice at 2 months of age; male mice developed PKD 9 months post Ift88 KO (at 11 months of age), while female Ift88 KO mouse kidneys had normal or rare renal cysts at this age (Hu et al., 2021). Further examination of male and female Ift88 KO mice at 2‐ and 9‐months post‐induction (induction at 2 months of age) revealed age‐ and sex‐specific blood pressure (BP) and urinary Na^+^ excretion (UNaV) phenotypes (Hu et al., 2021). In pre‐cystic Ift88 KO mice (2 months post‐induction), males had reduced BP and increased high salt diet‐induced UNaV compared to age‐ and sex‐matched controls, while female Ift88 KO mice had similar BP and UNaV as age‐ and sex‐matched controls. In Ift88 KO mice 9 months post‐induction, males manifested salt‐dependent hypertension associated with prominent cystic kidneys, while females had reduced BP compared to age‐ and sex‐matched controls while fed a high salt diet. Although intrinsic renal regulatory pathways associated with these phenotypes were investigated, alterations in nephron Na^+^ transporters and channels were not reported. Consequently, the current study examined the profile of nephron Na^+^ transporters and channels in Ift88 KO mice at 2‐ and 9‐months post‐induction and in their age‐ and sex‐matched controls.

## 2 METHODS

### 2.1 Animal care

All animal studies were conducted with the approval of the University of Utah Animal Care and Use Committee in accordance with the National Institutes of Health Guide for the Care and Use of Laboratory Animals.

### 2.2 Generation of inducible nephron specific Ift88 KO mice

Nephron‐specific Ift88 KO mice were generated as previously described (Hu et al., 2021). Floxed Ift88 mice with loxP sites flanking exons 4 to 6 of the *Ift88* gene were bred with C57/BL6 mice containing the Pax8‐reverse tetracycline transactivator (rtTA) (Pax8 promoter‐rtTA confers nephron‐specific targeting) and LC‐1 transgenes (the LC‐1 transgene contains doxycycline/rtTA‐inducible Cre recombinase and luciferase) (Lakshmipathi et al., 2020). All mice were homozygous for the loxP‐flanked *Ift88* gene and hemizygous for Pax8‐rtTA and LC‐1 transgenes. Doxycycline (DOX, 2 mg/ml) was given in 2% sucrose drinking water to 2‐month‐old mice for 12 days (Ift88 KO). Littermates of the same genotype and sex, but without DOX treatment, were used as controls. Control and Ift88 KO mice aged 4 months (2 months post‐DOX) and 11 months (9 months post DOX) (1:1 male:female) were studied.

### 2.3 Genotyping and determination of *Ift88* gene recombination

Genotyping PCR on tail DNA was performed using the Ift88 forward 5’‐GACCACCTTTTTAGCCTCCTG‐3’ and reverse 5’‐GAATAGTGGCAATTCTGGCTC‐3’ primers which yielded a 260 bp product from the floxed *Ift88* gene and a 220 bp product from the wild‐type allele; Pax8‐rtTA forward 5′‐CCATGTCTAGACTGGACAAGA‐3′ and reverse 5′‐CATCAATGTATCTTATCATGTCTGG‐3′ primers yielded a 600 bp product; and LC‐1 forward 5′‐TCGCTGCATTACCGGTCGATGC‐3′ and reverse 5′‐CCATGAGTGAACGAACCTGGTCG‐3′ primers yielded a 480 bp product.

### 2.4 Western analysis

Ift88 KO (2‐ and 9‐months post DOX) and age‐ and sex‐matched control mice were fed 3 days of a normal Na^+^ diet (0.3% Na^+^, Micro‐stabilized Rodent Liquid Diet LD101, TestDiet, St. Louis, MO) and 3 days of a high Na^+^ diet (3.2% Na^+^, TestDiet LD101 with added NaCl). Whole kidneys were removed at the end of 3 days, weighed, homogenized, protein isolated and immunoblotting performed. Samples were homogenized in ice‐cold buffer containing 250 mM sucrose, 10 mM triethanolamine, pH 7.6 with 100 μg/ml PMSF, 200 mM sodium orthovanadate, 200 mM sodium fluoride, and 1 mg/ml leupeptin. Total protein concentration was measured using the bicinchoninic acid protein assay kit (Pierce, Waltham, MA). Samples were diluted with Laemmli buffer, heated at 65°C for 15 min, and stored at −80°C in aliquots to avoid repeated freeze/thaw. Proteins were separated using a 4–12% bis‐tris mini gel (Invitrogen, Carlsbad, CA) and transferred onto a PVDF membrane. Membranes were blocked with 5% nonfat dry milk and 3% bovine serum albumin in tris buffered saline with tween (TBST) for 1 h at room temperature. Membranes were incubated with specific primary antibodies overnight at 4°C except for 1 h incubation with anti‐phospho‐NHE3 antibody. After washing with TBST, membranes were incubated with horseradish peroxidase‐conjugated secondary antibodies for 1 h at room temperature.

Primary antibodies were as follows (Table 1): ENaC‐α, ‐β, and ‐γ, GAPDH, total and phosphorylated NHE3, total and phosphorylated (T^53^) Na^+^/Cl^−^ cotransporter (NCC), and total Na^+^/K^+^/2Cl^−^ cotransporter (NKCC2). Secondary horseradish peroxidase‐conjugated antibodies were goat anti‐mouse IgG (1:2000, Abcam) and goat anti‐rabbit IgG (1:2000, Abcam). Horseradish peroxidase was visualized using the Advance ECL System with ProSignal Dura ECL Reagent (Genesee Scientific, San Diego, CA) or Amersham ECL Primer Western Blotting Detection Reagent (GE Healthcare, Piscataway, NJ). Images were obtained and quantified by ImageLab (Bio‐Rad, Hercules, CA). All antibodies were initially tested for linearity by loading 1, 2.5, 5,10, 20, 40, and 60 µg of protein; linear results were obtained for all antibodies between 2.5 and 10 µg, so 7.5 µg of protein was loaded into each lane for all experiments. Normalizing to GAPDH was performed.

**TABLE 1:** Primary antibodies used for western analysis

Description	Size (kDa)	Species source	Dilution	Company	Reference
Glyceraldehyde phosphate dehydrogenase	37	Rabbit	1:1000	2118, Cell Signaling, Danvers, MA	Pang et al. (2021)
Epithelial Na channel‐α	85	Rabbit	1:1000	SPC‐403, StressMarq, Victoria, BC	Veiras et al. (2021)
Epithelial Na channel‐β	85	Rabbit	1:1000	SPC‐404, StressMarq	Wu et al. (2020)
Epithelial Na channel‐γ	85	Rabbit	1:1000	SPC‐405, StressMarq	Song et al. (2020)
Na/Cl co‐transporter	150	Rabbit	1:1000	David Ellison, Oregon Health Sciences University	Bostanjoglo et al. (1998)
Na/Cl co‐transporter (phospho‐T^53^)	150	Rabbit	1:1000	P1311‐53, PhosphoSolutions, Aurora, CO	Duan et al. (2021)
Na/H exchanger 3	80	Mouse	1:1000	MAB3136MI, Fisher Scientific, Waltham, MA	Dos Santos et al. (2004)
Na/H exchanger 3 (phospho‐S^552^)	80	Mouse	1:1000	SC‐53962, Santa Cruz	Nelson et al. (2021)
Na/K/2Cl co‐transporter	160	Rabbit	1:1000	Mark Knepper, NHLBI	Kim et al. (1999)

### 2.5 Statistical analysis

All experiments involved *N* = 4 for each data point. The Student's *t*‐test was used to compare differences in protein expression between Ift88 KO and control mice of the same sex and fed the same diet. All analysis was performed using GraphPad Prism 9 software. The criterion for significance was *p* < 0.05.

## 3 RESULTS

### 3.1 Ift88 KO model

Nephron‐specific Ift88 KO mice have been previously described (Hu et al., 2021), including confirmation of virtually complete abolition of nephron Ift88 mRNA and nephron cilia in both sexes. As discussed earlier, male Ift88 KO mice 2 months post‐DOX had lower BP and higher salt‐induced natriuresis compared to male controls, while female Ift88 KO mice had similar BP and Na^+^ excretion compared to female controls (Hu et al., 2021). At 9 months post DOX, male Ift88 KO mice had polycystic kidneys and elevated BP compared to male controls, while female Ift88 KO mice had very rare renal cysts and reduced BP compared to female controls (Hu et al., 2021). While these are not new results, they are described again in the Results section to provide context for the western analysis studies.

### 3.2 Renal Na^+^ transporter and channel expression 2 months post Ift88 KO

**FIGURE 1:**
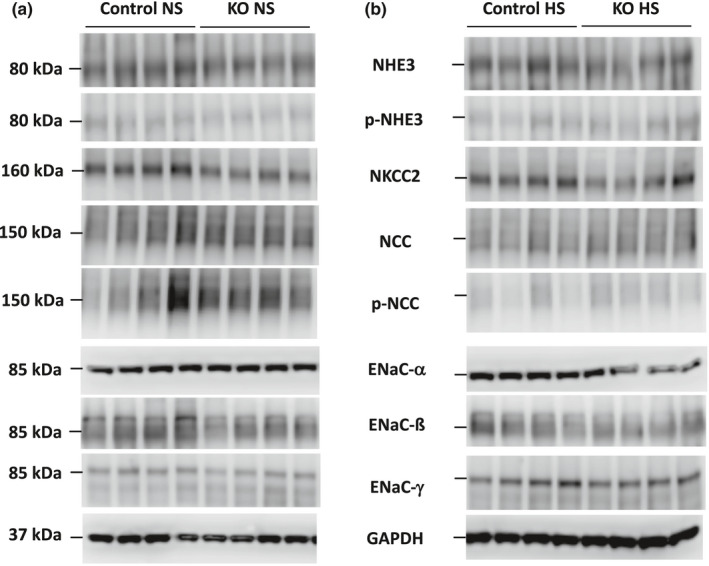
Western blots of Na^+^ transporters and channels and GAPDH in 2 months post DOX female Ift88 KO and control mice fed a normal (a) or high (b) salt diet

**FIGURE 2:**
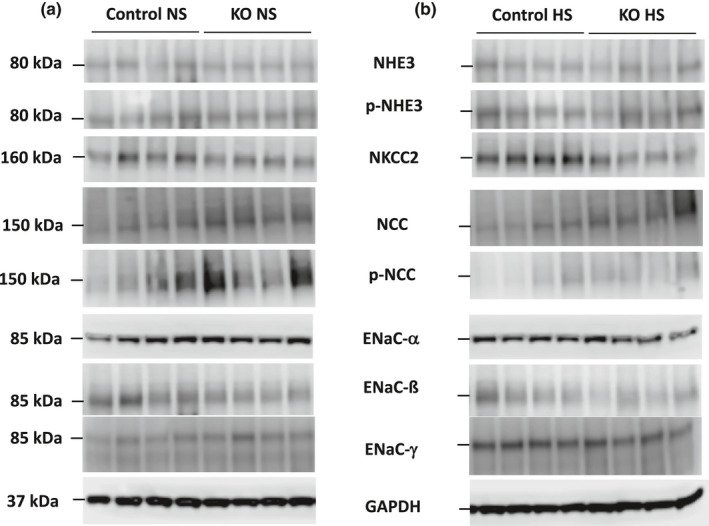
Western blots of Na^+^ transporters and channels and GAPDH in 2 months post DOX male Ift88 KO and control mice fed a normal (a) or high (b) salt diet

**FIGURE 3:**
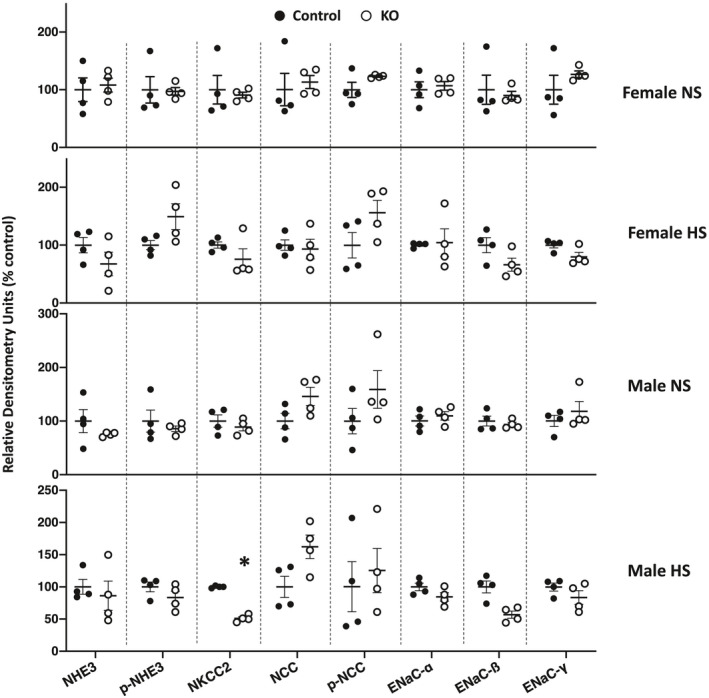
Western analysis of female normal and high salt fed, and male normal and high salt fed, Ift88 KO (2 months post DOX) and control mouse kidneys. All westerns for high salt diets were obtained on day 3 of high salt intake. *N* = 4 each data point. *p < 0.05 KO vs. control on the same diet

Western analysis of the major kidney Na^+^ transporters and/or channels revealed no differences in ENaC isoforms, total or phosphorylated NHE3, total or phosphorylated (T^53^) NCC, or total NKCC2 between female Ift88 KO 2 months post‐DOX and age‐matched control female mice fed a normal or high Na^+^ diet (Figures 1 and 3). Male Ift88 KO 2 months post‐DOX mice fed a normal salt diet also had no differences from age‐matched male control mice in any of the above transporters and/or channels (Figures 2 and 3). In contrast, high salt fed male Ift88 KO mice 2 months post‐DOX had reduced total NKCC2 compared to age‐ and diet‐matched male control mice (Figures 2 and 3). There were no significant differences in the other Na^+^ transporters/channels between high salt fed male Ift88 KO 2 months post‐DOX and age‐ and diet‐matched control male mice (Figures 2 and 3). Note that phosphorylated (T^96/101^) NKCC2 was not measured due to a recent report demonstrating that currently available anti‐phosphorylated (T^96/101^) NKCC2 antibodies cross react with phosphorylated NCC specifically in C57BL/6 mice (Moser et al., 2021).

### 3.3 Renal Na^+^ transporter and channel expression 9 months post Ift88 KO

**FIGURE 4:**
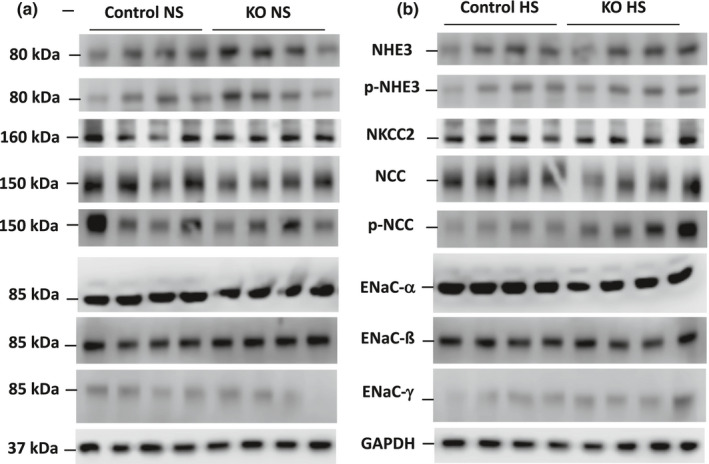
Western blots of Na^+^ transporters and channels and GAPDH in 9 months post DOX female Ift88 KO and control mice fed a normal (a) or high (b) salt diet

**FIGURE 5:**
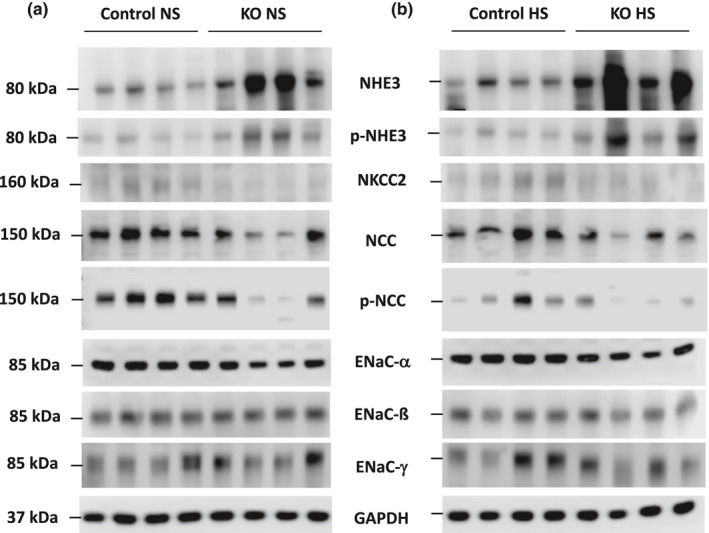
Western blots of Na^+^ transporters and channels and GAPDH in 9 months post DOX male Ift88 KO and control mice fed a normal (a) or high (b) salt diet

**FIGURE 6:**
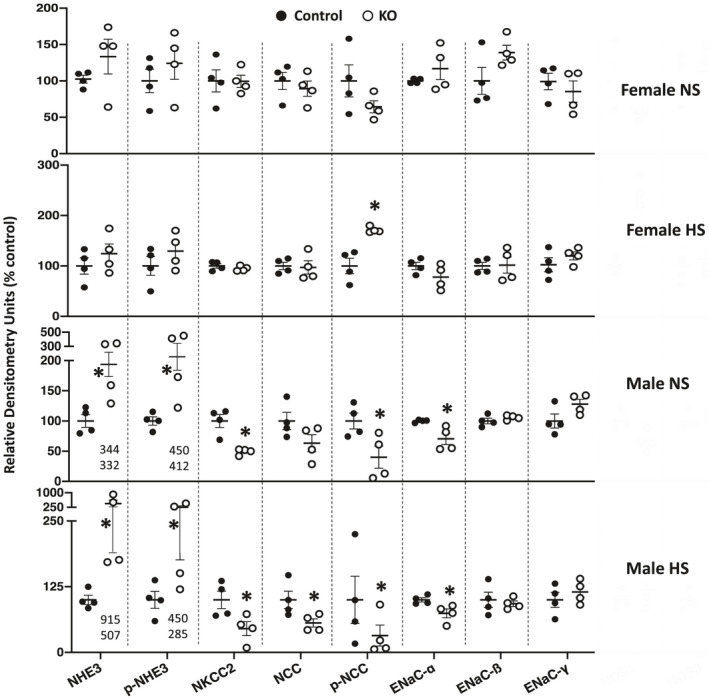
Western analysis of female normal and high salt fed, and male normal and high salt fed, Ift88 KO (9 months post DOX) and control mouse kidneys. All westerns for high salt diets were obtained on day 3 of high salt intake. *N* = 4 each data point. The numbers shown for NHE3 and pNHE3 for male normal and high salt diets indicate the two highest values for Ift88 KO mice in each condition since these are harder to ascertain due to Y‐axis compression. *p < 0.05 KO vs. control on the same diet

Western analysis revealed that female Ift88 KO mice 9 months post‐DOX had no alterations in any of the renal Na^+^ transporters and channels compared to age‐ and diet‐matched control female mice regardless of salt intake with the exception that phospho‐NCC was elevated in high salt fed female Ift88 KO mice compared to age‐matched high salt fed female control mice (Figures 4 and 6). Normal and high salt fed male Ift88 KO mice 9 months post‐DOX kidneys had markedly elevated total and phosphorylated NHE3 expression compared to age‐ and diet‐matched control male mice; this was accompanied by relative reductions (compared to age‐ and diet‐matched male control mice) in ENaC‐α, phospho‐NCC and total NKCC2 protein expression (Figures 5 and 6).

## 4 DISCUSSION

The current study made the following key observations: (1) Relatively hypotensive pre‐cystic Ift88 KO male mice have reduced renal NKCC2 expression; and (2) hypertensive Ift88 KO males with polycystic kidneys have increased total and phosphorylated NHE3 expression associated with decreased NKCC2, NCC, and ENaC protein levels. In contrast, age matched female Ift88 KO mice had minimal alterations in Na^+^ transporters or channels.

The physiological role (in the absence of renal cysts) of nephron cilia in BP control and renal Na^+^ transporter and/or channel expression/activity is not well understood. Previous studies, albeit *in vitro*, have shown that cilia are involved in tubule luminal mechanosensation, potentially modulating ENaC and NHE expression/activity (Olteanu et al., 2006, 2012). Thick ascending limb‐specific disruption of cilia (using NKCC2‐Cre to achieve Ift88 KO) caused salt‐sensitive hypertension and enhanced tubuloglomerular feedback in male mice without cysts (females were not studied and renal Na^+^ transporters were not measured) (Song et al., 2017), suggesting that thick ascending limb cilia disruption, independent of cysts and at least in males, elicits hypertension. In contrast, as previously discussed, we found that pre‐cystic male mice with nephron wide Ift88 KO had reduced BP (Hu et al., 2021); the current study found reduced salt loading dependent NKCC2 expression in these mice. Further, mice (sex‐dependency was not evaluated) with nephron specific *Pkd1* gene disruption have reduced BP and decreased NKCC2 expression before cyst formation (Lakshmipathi et al., 2020), although it must be noted that polycystin‐1 biological actions can be distinct from those of cilia. The reasons for the different results between the NKCC2‐Cre and the nephron‐wide KO Ift88 KO studies are speculative; one notable difference is that NKCC2‐Cre is expressed during embryogenesis, while the nephron‐wide KO was induced during adulthood. Ultimately, it will be very interesting to examine the effects of induced thick ascending limb specific Ift88 KO during adulthood should those mice become available.

While cyst‐associated hypertension is expected, these were the first studies, to our knowledge, to profile nephron Na^+^ channel/transporter expression in cystic kidneys. Interestingly, total and phosphorylated renal NHE3 was elevated in cystic male Ift88 KO mice; the cause of this was not examined, however, one possibility is intrarenal renin‐angiotensin system activation due to cyst compression of the renal vasculature leading to angiotensin II‐augmented NHE3 expression (Chapman, 2007). Another possible factor is the reduced urinary NO excretion in male Ift88 KO mice cystic kidneys observed during high salt intake (Hu et al., 2021). Notably, NKCC2, phospho‐NCC, and ENaC‐α expression were all downregulated in male Ift88 KO cystic kidneys raising the possibility of a compensatory response to enhanced NHE3 activity.

The reduced BP previously reported (Hu et al., 2021) during the high salt diet in female Ift88 KO mice 9 months post DOX was not associated with detectable changes in renal Na^+^ channels/transporters with the surprising exception of elevated phospho‐NCC expression during high salt intake; the reasons for and significance of this latter finding are uncertain. These findings may reflect the fact that the BP reduction was quite small in these females (Hu et al., 2021)—perhaps they go on to develop greater BP reduction, frankly evident Na^+^ excretion and broader Na^+^ transporter/channel changes.

The reasons for the marked sex difference in Ift88 KO mouse kidney cystogenesis, BP and Na^+^ transporter/channel expression are unknown. Males are more susceptible to renal cystogenesis as has been demonstrated in the current study's model (Hu et al., 2021) and previously in Han:SPRD (mutation in the *Anks6* gene) rats, male PCK (mutation in *Pkhd1* gene) rats, male Jck (mutation in the *Nek8* gene) mice, and in humans with PKD (Cornec‐Le Gall et al., 2017; Johnson & Gabow, 1997; Lager et al., 2001; Schrier et al., 2014; Smith et al., 2006; Stewart, 1994; Stringer et al., 2005). One obvious possibility relates to gonadal hormones: Testosterone can promote renal vasoconstriction, fibrosis, and inflammation (Lima‐Posada & Bobadilla, 2021).

In conclusion, nephron Ift88 KO causes male‐specific changes in renal Na^+^ transporters and/or channels in both the pre‐cystic and cystic states. Whether these changes, including reduced NKCC2 expression in pre‐cystic Ift88 KO mice and increased NHE3 expression in cystic male Ift88 KO mice, are responsible for the observed relative hypotensive and hypertensive phenotypes, respectively, remains to be determined.

## CONFLICT OF INTEREST

None.

## AUTHOR CONTRIBUTION

Chunyan Hu and Donald E. Kohan designed the study; Chunyan Hu, Jayalakshmi Lakshmipathi, and Deborah Stuart carried out the experiments; Chunyan Hu, Jayalakshmi Lakshmipathi, Deborah Stuart, and Donald E. Kohan analyzed the data, Chunyan Hu, Jayalakshmi Lakshmipathi, and Donald E. Kohan made the figures; Chunyan Hu and Donald E. Kohan drafted and revised the paper; all authors approved the final version of the manuscript.
